# Computational Immunology Meets Bioinformatics: The Use of Prediction Tools for Molecular Binding in the Simulation of the Immune System

**DOI:** 10.1371/journal.pone.0009862

**Published:** 2010-04-16

**Authors:** Nicolas Rapin, Ole Lund, Massimo Bernaschi, Filippo Castiglione

**Affiliations:** 1 Biotech Research and Innovation Centre and Bioinformatics Centre, University of Copenhagen, Copenhagen, Denmark; 2 Center for Biological Sequence Analysis, Department of Systems Biology, Technical University of Denmark, Lyngby, Denmark; 3 Institute for Computing Applications, National Research Council, Rome, Italy; Dana-Farber Cancer Institute, United States of America

## Abstract

We present a new approach to the study of the immune system that combines techniques of systems biology with information provided by data-driven prediction methods. To this end, we have extended an agent-based simulator of the immune response, C-ImmSim, such that it represents pathogens, as well as lymphocytes receptors, by means of their amino acid sequences and makes use of bioinformatics methods for T and B cell epitope prediction. This is a key step for the simulation of the immune response, because it determines immunogenicity. The binding of the epitope, which is the immunogenic part of an invading pathogen, together with activation and cooperation from T helper cells, is required to trigger an immune response in the affected host. To determine a pathogen's epitopes, we use existing prediction methods. In addition, we propose a novel method, which uses Miyazawa and Jernigan protein–protein potential measurements, for assessing molecular binding in the context of immune complexes. We benchmark the resulting model by simulating a classical immunization experiment that reproduces the development of immune memory. We also investigate the role of major histocompatibility complex (MHC) haplotype heterozygosity and homozygosity with respect to the influenza virus and show that there is an advantage to heterozygosity. Finally, we investigate the emergence of one or more dominating clones of lymphocytes in the situation of chronic exposure to the same immunogenic molecule and show that high affinity clones proliferate more than any other. These results show that the simulator produces dynamics that are stable and consistent with basic immunological knowledge. We believe that the combination of genomic information and simulation of the dynamics of the immune system, in one single tool, can offer new perspectives for a better understanding of the immune system.

## Introduction

The immune system, due to its very complex nature, is one of the most challenging topics in biology. Its study often relies on *in vivo* or *in vitro* animal models, mathematical models, or computational (*in silico*) models. Recent advances in the field of bioinformatics have provided a number of techniques for processing and integrating the explosion of data that has been produced during the rise of genomics, which has also improved our ability to predict the molecular specificities of the immune system (for a review see e.g., [1]). A number of mathematical models based on either differential equations or interacting discrete entities (*agents*) have also been proposed to describe various aspects of the immune system. However, most of the existing simulation-based approaches resort to oversimplified models of molecular interactions, because detailed quantitative data, needed for a more realistic representation, were not always available.

The goal of the present work is to present a novel approach for the study of the immune system, combining a mesoscopic scale simulator of the immune system [2] with a set of machine learning techniques for molecular-level predictions of major histocompatibility complex–peptide binding interactions [3]–[6], linear B cell epitope discovery, as well as a more general protein–protein potential estimation [7]. More specifically, the computational model belongs to an *agent-based* class, whereas the prediction of epitopes relies on machine learning techniques, such as Neural Networks (NN).

The paper is organized as follows: After an introduction to the fundamental mathematics required for modeling the immune system, we present results of simulations whose aim is to test the correctness the new tool. We concludes the paper with a perspective on the future of this work. Finally, the materials and methods section describes the bioinformatics tools used for predicting the interactions among the entities involved in the immune response, including a description of how they are incorporated into the mesoscopic C-ImmSim simulator.

### *In silico* models of the immune system

The immune system can be viewed as a classic system of coupled components, with birth, death, and interaction elements. The most common modeling approach utilizes systems of either Ordinary or Partial Differential Equations (ODE and PDE, respectively) that directly describe the evolution of global quantities or populations over time [8]. In immunology, these quantities could be, for instance, the total concentration of viral particles or cell counts. ODE- and PDE-based models enable a model to use well-established analytical and numerical techniques, but they potentially oversimplify the system: an entire population of discrete entities is described by a single continuous variable. Mathematical models based on differential equations have proved very useful. The study of the evolution of HIV into AIDS, for instance, has been modeled with the purpose of predicting the effects of specific treatments [9]–[12], and predicting certain aspects of disease progression [13]–[23].

Each entity (e.g., a cell) is individually represented by an *agent*, and the interactions among agents are defined by a set of rules that can have stochastic components. The rules reflect the current knowledge in immunology, but they can also be defined *ad hoc* to test new hypotheses regarding the operation of the immune system.

One of the first attempts to define a detailed agent-based model of immunological mechanisms was the work of Celada and Seiden [2], [24], [25]. Their goal was to capture the dynamics of the immune system, as much as possible, and to perform experiments *in silico*. Along similar lines, a study of the thymus has been carried out [26]. This approach provided important insights into the regulation of positive and negative selection and into the dynamics of the production of the TCR repertoire in the thymus. More recently, we have developed specialized versions of the Celada-Seiden model to study HIV-1 infection, EBV infection, hypersensitivity reactions, and cancer immunoprevention (described, respectively, in [27]–[30]). Recentely, another implementation of the same model has been used to study cross reactivity and heterologous memory [31].

C-ImmSim, the simulator that implements our version of the Celada-Seiden model, is a flexible tool that can be used for the study of a number of different immunological processes. The original model used bit strings to represent the *receptors* of biological entities.

### Related works

Recently, there has been renewed interest in modeling the immune system by means of agent-based models.

Simmune [32] aims at being a flexible platform for the simulation of any immunological process. It is more of a modeling technique and a language for the description of models than a specific model. Simmune is based on a particular representation of particle interactions that can be used to create detailed models of the immune system. The particles live on a mesh, and their states are updated at discrete time-steps so that both time and space are discrete. Particles in Simmune can be in different states. Transitions among the states are probabilistic events triggered by the exchange of *messenger* particles having a limited range. The messenger field intensities are calculated by the integration of reaction-diffusion equations and typically include an activation threshold. A major advantage of Simmune is that it models both direct intercellular interactions (such as those between an antigen and a B cell) and interactions mediated by molecular messengers (such as lymphokines). It also supports spatial compartmentalization and communication conduits.

The Basic Immune Simulator (Bis) [33] is an agent-based model created to study the interactions among the cells of the innate and adaptive immune systems. Bis simulates basic cell types, mediators, and antibodies, and consists of three virtual spaces representing parenchymal tissue, secondary lymphoid tissue, and the lymphatic/humoral circulation. Bis translates mechanistic cellular and molecular knowledge regarding the innate and adaptive immune response and reproduces the immune system's complex behavioral patterns. It has been used both as an educational tool to demonstrate the emergence of those patterns and as a research tool to systematically identify potential targets for more effective treatment strategies for diseases processes, including hypersensitivity reactions, autoimmunity, and cancer.

Simisys [34] is a cellular automata-based method that allows the simulation of tens of thousands of cells. Both innate and adaptive components of the immune system are represented. Specifically, macrophages, dendritic cells, neutrophils, natural killer cells, B cells, T helper cells, complement proteins, and pathogenic bacteria are present in the model.

### Bit string models of immune diversity

A fundamental task of the immune system is to recognize and bind antigens by means of cell receptors. The binding mechanism is based on physical–chemical processes (short-range non-covalent interactions, hydrogen bonding, van der Waals interactions) [8].

The features that determine the binding among molecules [35] may be represented by a *shape-space*. Under the assumption that the shape-space can be described by means of 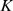 parameters, a point in this 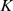-dimensional space specifies the generalized shape of a binding region. Oster and Perelson estimated that in order to be complete, the receptor repertoire should fulfill the following conditions: *i)* each receptor should recognize a set of related epitopes, each of which differs slightly in shape; *ii)* the repertoire size should be on the order of 10^16^ or larger; *iii)* at least one subset of the repertoire should be distributed randomly throughout the shape-space. Later, Farmer and co-workers [36] introduced the idea of using binary strings to represent the generalized shape of a receptor. To determine the degree of affinity between bit-strings, it is possible to resort to different string-matching criteria. For instance, by using a key–lock analogy, two binary strings have a high affinity if they complement each other, that is, when the two strings are lined up, every “0” in one string corresponds to a “1” in the other, and conversely. The bit string representation of antigen and cell receptor diversity was then adopted by a number of other authors [37]–[39], and has been the basis for the description of all molecular interactions in earlier implementations of the Celada-Seiden model.

### Previous version of C-ImmSim

The C-ImmSim model of the immune system response has been quite extensively described in [27], [40]. C-ImmSim was implemented in ANSI C language. In short, it consists of a three-dimensional (3D) stochastic cellular automaton in which the major classes of cells of both the lymphoid (T helper lymphocytes (Th), cytotoxic T lymphocytes (CTL), B lymphocytes, and antibody-producer plasma cells, PLB) and the myeloid lineage (macrophages (M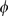) and dendritic cells (DC)) are represented. All these entities interact with each other according to a set of rules that describe the different phases of the recognition and response processes of the immune system against a pathogen.

C-ImmSim can be classified as a bit-string polyclonal lattice model. Bit-string refers to the way in which the molecules are represented, polyclonal indicates that the lymphocytes have genetic variation in their receptors, and lattice signifies that a discrete lattice is used to represent the space.

The model mainly represents a portion of a tertiary organ such as a lymph node, tonsil, or spleen. Tertiary organs are sites in which antigens are presented to immune cells. C-ImmSim simultaneously simulates three compartments that represent three separate anatomical regions found in mammals: (i) the bone marrow, where hematopoietic stem cells are simulated, which produce new lymphoid and myeloid cells; (ii) the thymus, where naïve T cells are selected to avoid auto-reactivity; and (iii) a tertiary lymphatic organ, such as a lymph node. The tertiary organ is the only compartment that is described geometrically, because it is mapped onto a 3D lattice. All interactions among cells and molecules take place on a lattice-site during each time step. The diffusion of entities at each time step models the physical spreading of molecules in the lymphatic organ.

A set of self peptides is used to define the “self” at the beginning of the simulation. Non-self is defined as everything else. Potential pathogens as well as cell receptors and MHC molecules (the HLA or Human Leukocyte Antigen), are represented as binary strings.

In the model, all cells are considered ‘active’ or ‘resting’. This means that naïve cells are not taken into account. Hence, all cells reaching the tertiary organ (the simulation space) are already mature. T-lymphocytes are exceptions, because they undergo thymic selection before entering circulation. The lymphocytes generated in the bone marrow have a high diversity with respect to their receptors, due to *alternative splicing*, which is somatic rearrangement of noncontiguous genomic V, J, and C regions, and sometimes *hypermutation*. We represent this phenomenon by assigning random bit-string receptors to every lymphocyte.

C-ImmSim incorporates the following working hypothesis or theories: *i)* the diversity of specific elements [41]–[43]; *ii)* antigen processing and presentation [44]–[47]; *iii)* MHC restriction [48]; *iv)* cell–cell cooperation [49], [50]; *v)* maturation of the response and memory [51], [52]; *vi)* clonal selection by antigen affinity ([53]); *vii)* thymus education of T lymphocytes (clonal deletion theory, [54]); *viii)* hypermutation of antibodies; *ix)* Hayflick limit (T cell replicative senescence [55]–[58]); *x)* Ag dose-induced tolerance (anergy) in B cells [59], [60].

*xi)* T cell anergy [61];

*xii)* Matzinger's danger signals [62];

*xiii)* Idiotypic Network theory [63]. Further information can be found in the literature.

## Results

In the remainder of this section, we describe some numerical experiments that were designed with the goal of assessing the soundness of the simulator. The average execution time was around three hours on a 2.4 GHz Opteron processor. In terms of memory consumption, the simulation of a 10 microliter volume requires 1 GB of RAM.

### Immunization experiment

In this experiment, we reproduce the typical immunization process by injecting an immunogenic protein at two subsequent instants in time. The actual AA string used as an antigenic molecule is the gag molecule from HIV-1.

**Figure 1:**
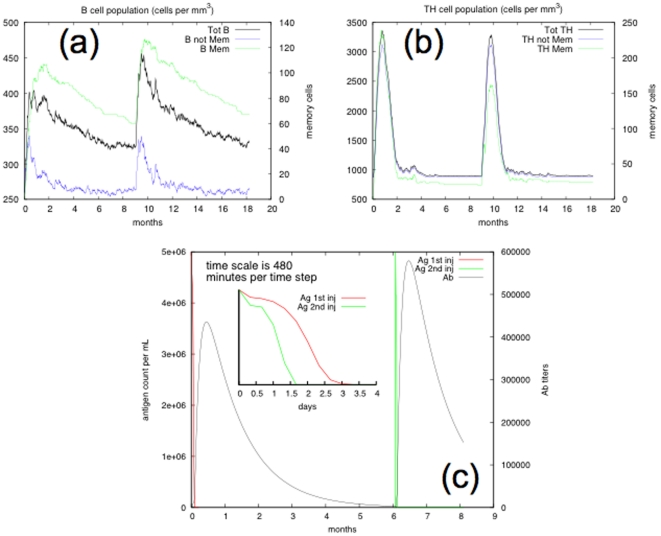
Simulation of an immunization experiment. B cell (panel a) and CD4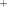 T cell (panel b) population during a typical immunization experiment. An immunogenic molecule is injected at time zero and after six months. In both plots, the total number of lymphocytes along with the immune memory compartment are shown. Panel (c) shows that the secondary response eliminates the antigen on a shorter timescale due to the presence of memory cells ready to react.

The antigen is injected at time zero, then again six months (of simulated time) later. The system develops a typical primary and secondary immune response with a significant increase in memory lymphocytes, as shown in Figure 1. Panel (a) and panel (b) show, respectively, the cell counts of B and T helper lymphocytes in a cubic millimeter. The immunological memory is developed during the first response. Therefore, the second response is much more rapid, as can be seen in the inset plot of panel (c), which shows the time the immune system takes to clear the antigen. The same panel also shows the humoral response in terms of antibody titers (arbitrary scale). In summary, the dynamics are consistent with a realistic immunization process, because they show a faster secondary response due to the development of long-lasting memory.

### Immunodominance or affinity maturation

In this experiment, we test the emergence of one or more dominating clones of lymphocytes in the situation of chronic exposure to the same immunogenic molecule. In other words, we check if the system reproduces the phenomenon of *affinity maturation*. To mimic chronic exposure to a pathogen, we repeatedly inject a certain amount of the *HIV/gag* protein (for example) throughout the simulation period.

**Figure 2:**
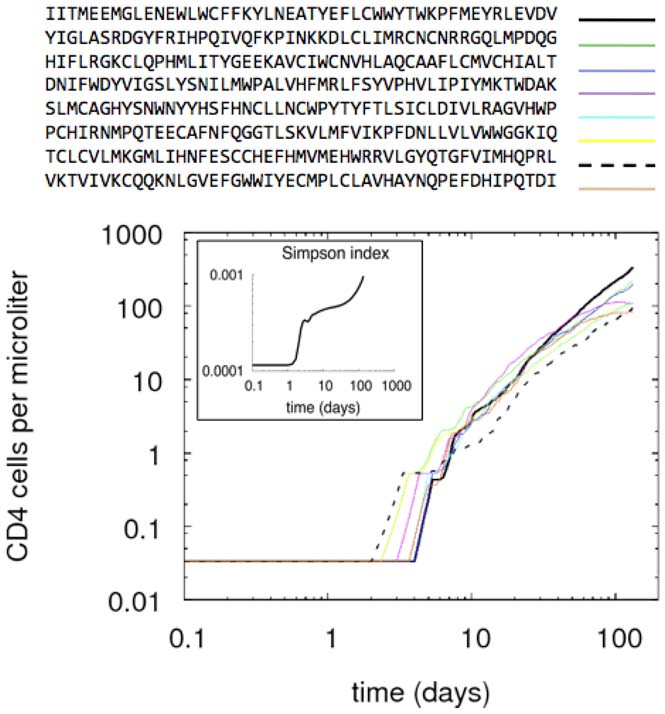
The T helper lymphocyte count for the most representative clones that are involved in the immune response, e.g., those that are antigen-specific. In the inset of the same panel, we plot the Simpson index.

The system responds by mounting a specific immune response from the beginning of exposure. Then, as the simulation proceeds, higher affinity clones overtake the original clones with respect to expression levels, eventually proliferating at higher levels than any other. This is shown in Figure 2, in which the lymphocyte T helper count for the top-ranked clones is shown alongside the specific TCRs. Note, in particular, that the dashed line corresponds to the first emerging clone, and the continuous line shows a later-appearing clone with a better affinity that overcomes the first clone. In the inset plot of the same figure, we show the Simpson index 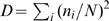, where 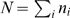 and 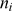 is the count of the clone with specificity 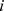. The increase of the index 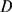 over time indicates the emergence of a dominating clone (i.e., the bigger the value of 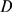, the lower the diversity).

### Homozygote vs heterozygote

There are reasons to believe that the “time to AIDS” in HIV-infected patients is related to the haplotype homozygosity [64]. Individuals that bear a higher diversity in their MHC have slower progression to AIDS than those with lower diversity. In the analysis carried out by Carrington [64], individuals carrying heterozygosity for HLA-A, HLA-B, or HLA-C each showed a longer AIDS-free period compared to their homozygote locus counterparts. Here, we simulate the situation and compare the time to clear a given antigen (not specifically the HIV-1) for individuals with full heterozygosity and individuals with homozygosity for their MHC loci. The heterozygote haplotype is a full heterozygote, meaning that we allow all possible loci to be different. The homozygotes bear one A- allele, one B- allele, and one DR- allele. The following set of MHCs have been used, following the article by Hoof *et al.*, in which MHC alleles are ranked based on observed viremia levels in HIV-I-infected patients [65]: homozygote genotype: A
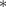
0201, B
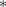
5304, DRB3
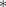
0302; heterozygote genotype: A
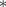
0201, A
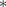
0301, B
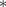
5304,B
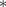
5309, DRB3
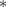
0302,DRB5
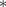
0202.

**Figure 3:**
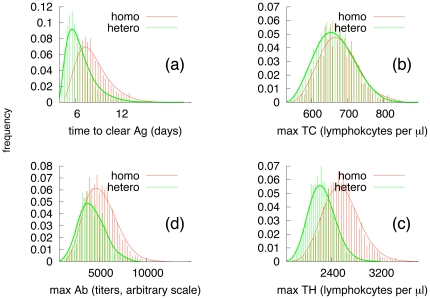
Immune response over 500 different simulations. Panel (a) shows the distribution of the time to clear the antigen in five hundred simulations with different random seeds. Panels (b), (c), and (d) show that the immune effort calculated as the maximum number of cells (both CTLs, CD4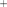 T-cells) during the immune response, is higher for the homozygote type.

The antigen used encompassed all proteins from the Flu influenza A serotype H1N1 (genome id: HU13275) in the flu genome database (www.flugenome.org). For demonstration purposes, we assume that the virus does not mutate. The results of simulations, shown in Figure 3, indeed show that the speed of antigen clearance is faster for simulations with a heterozygote haplotype. It is also worth noting that the immune effort calculated as the number of cells (both CTLs and CD4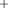 T-cells) during the immune response is higher for the homozygote-type, consistent with the fact that the homozygote immune response is poorer and, therefore, allows the virus to grow to higher numbers before it is cleared. The opposite situation holds for the heterozygote immune system, which optimally clears the virus faster and with less effort.

## Discussion

We have presented an integrated multi-level model that describes the immune system response at the mesoscopic level and, at the same time, takes into account the recognition mechanisms among molecules by means of prediction tools based, in part, on well known techniques for epitope discovery.

We have tested the simulator in typical immunization experiments, in which the immune system develops memory. We have shown that the system develops affinity maturation against “chronic” antigenic peptides, and we have also shown that heterozygosity helps the immune system to cope with the diversity of pathogens. These results show that the simulator produces dynamics that are consistent with previous versions of C-ImmSim. Additionally, the simulation extends those results by using AA strings, adding a considerable quantity of information. This feature precisely describes the added value of a tool of this kind.

The novelty and the power of our approach lie in the use of a combination of two levels of description to study the immune response by means of computer simulation. The first level is a mesoscopic agent-based model representing cooperating cells that mount an immune response. The second level is a set of molecular binding prediction methods that are used to compute the binding affinity of the molecules represented in the agent-based model. The combination of these tools allows us to perform *in silico* immunization experiments with specific real-world proteins and could help to speed up drug design or clinically oriented research.

The system also provides a framework for testing various prediction methods, because the two levels of description, molecular interactions and cellular interactions, have purposefully been kept separate in the computer code. This implies that a novel method for predicting B cell epitopes could, for example, be easily inserted into the simulator with minimal programming effort, and the consequences could be immediately analyzed by looking at the resulting immune dynamics.

Further work will focus on optimizing the procedures and finding better algorithms for prediction of B cell epitopes. The Miyazawa-Kernigan potential, which we have used to predict the binding affinity between generic AA strings, can also be replaced with a more accurate prediction method, should one become available. The proposed architecture has been developed with consideration for the issue of upgradability and modularity so that new prediction methods can be easily inserted and used.

To conclude, we believe this tool has the potential to “grow” by acquiring precision, becoming a more and more useful prediction tool in immunological research, in which *in vivo* or *in vitro* studies of drugs and their effects on the immune response are too difficult or expensive (either in terms of money or time) to carry out. For example, it is possible to investigate why one particular epitope of a given antigen is more immunogenic than another. Does the uptake by the APCs determine this quality due to differences in presentation on MHCs, or to differences in ligation through immuno receptors? It is also possible to combine this kind of analysis with simulations of different types of pathogenic behavior and to study the cross-reactivity in the development of the flu vaccine to select for the best combination of known viral peptides to be used in order to achieve better protection. These are just a few possible works that we are planning to pursue in the near future.

## Materials and Methods

At the molecular level, a key step for the simulation of the immune response is the prediction of immunogenicity. Only the immunogenic parts of an invading pathogen will trigger an immune response in the affected host. Those parts are called epitopes and are pathogen-dependent.

### Molecular binding

In the specific context of the pathogen-induced immune response, one distinguishes between B cell and T cell epitopes. B cell epitopes are recognized by immunoglobulins, also known as antibodies. The immunogenic parts are often located on the surface of pathogenic proteins, because they have to be accessible for binding. The epitopes mostly consist of discontinuous blocks of the antigen sequence, i.e., sequence segments that are distantly separated in the protein sequence and are brought into proximity upon folding into tertiary or quaternary structures. The binding of a B cell epitope to a B cell receptor (BCR, an immunoglobulin covalently attached to the B cell surface) augmented by T helper cell induction triggers the differentiation of naïve B cells into antibody-secreting plasma cells that make up the humoral immune response. T cells can be divided into T helper cells (TH) and cytotoxic T lymphocytes (CTL or TC). T helper cells act as mediators between antigen presenting cells (APC) and plasma cells, and, therefore, assume a central role in the immune response. CTLs kill infected host cells by means of cytotoxic effector molecules that are released upon recognition of a complex on the surface of the infected cells. The complex consists of an epitope and an MHC class I molecule.

CTL epitopes are generated from cytosolic proteins. These peptides result from the antigen processing pathway that involves degradation by the proteasome, transport into the endoplasmic reticulum via the transporter associated with antigen processing (TAP), and presentation by MHC class I molecules. This processing takes place in all cells containing a nucleus. The MHC class II molecules, on the other hand, are produced only by APCs, which include dendritic cells, macrophages, and B cells. Epitope binding to MHC class II molecules are generated from internalized proteins that are degraded in acidified endocytic vesicles.

### Prediction methods

The immune system recognizes pathogens by means of their epitopes. As such, a protein belonging to a pathogen can be seen as a collection of parts that are either epitopes or non-epitopes. The binding strength of an epitope to a cell's receptor is one of the factors that determines the activation and strength of the immune response. For the last several years, we have developed computational methods that can predict T cell epitopes [3], [4], [6] or B cell epitopes [66], [67] in protein sequences. Although the neural networks for MHC prediction, developed in [3], [4], [6], seem to outperform other networks and methods [68], [69], it should be noted that these methods are not perfect. They cannot always provide the same level of accuracy as experimentally-generated data across all MHC alleles. Moreover, we assume that a peptide bound on the surface of an MHC molecule always triggers the immune system, which is not necessarily the case [70].

By implementing protein sequence-based representations for both the host and the pathogen, we may obtain a patient-specific genomic model capable of making specific predictions for different host/antigen genotype combinations.

Until now, C-ImmSim worked by using algorithms that represent the biological complexity using bit strings. If protein sequences rather than bit strings are used, different methods, such as Neural Networks, are needed to predict binding. The switch from bits to amino acids (AA) requires new algorithms to compute the affinity among strings. Because C-ImmSim is an agent-based model, every agent (e.g., any cell), along with its interactions, is individually simulated. This level of representation produces millions of bindings in a typical simulation. For this reason, we developed a new, fast *Position Specific Scoring Matrix* (PSSM)-based method, with minimal sacrifices with respect to the prediction of performance.

To assess the predicting power of the matrices, a large set of quantitative peptide MHC binding data were downloaded from the IEDB database [71]. The dataset consists of 6,533 peptides and covers 33 HLA- A and HLA-B human alleles. The PSSMs were calculated as described above, using the original NetMHCpan method trained only on human data. None of the peptides in the evaluation set were included in the training set. For each allele, the predictive performance of the corresponding PSSM was evaluated in terms of the Pearson's correlation between the log-transformed [72] IC50 value and the summed PSSM prediction score. Although the NetMHCPan method almost systematically outperforms the PSSM, the use of the latter in C-ImmSim is justified by the gain in computational speed.

The matrix method we employ has, on average, a Pearson's correlation coefficient of 0.56 with respect to experimental data, whereas the original NN performance was 0.62 (see Table 1) [73].

**Table 1:** Comparison of the predictive performance of the PSSM and NetMHCpan methods.

Allele	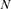	PSSM	NetMHCpan
A0101	446	0.705	0.789
A0201	442	0.641	0.724
A0301	329	0.584	0.638
A1101	217	0.646	0.684
A2301	329	0.553	0.568
A2402	367	0.519	0.527
A2403	111	0.592	0.653
A2601	428	0.747	0.821
A2902	329	0.500	0.520
A3002	329	0.539	0.473
A3101	224	0.629	0.759
A3301	224	0.561	0.700
A6801	224	0.632	0.780
B0801	119	0.481	0.543
B1501	114	0.336	0.424
B3901	106	0.445	0.508
B4001	230	0.679	0.733
B5801	102	0.389	0.435
Average	6533	0.560	0.620

The columns give the allele name, number of peptide data points 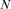, and the performance of the PSSM and NetMHCpan methods, respectively.

One of the major requirements for the integration of the prediction methods with an agent-based simulator is the development of tools that calculate the stability of molecular complexes.

Because there is no general method that can be used to predict if, for example, a TCR will interact with any given MHC–peptide complex, we have used the Miyazawa-Jernigan residue–residue potential [74] to score the strength of the interaction.

In the following sections, we present the implementation and combination of each of these processes. For a better understanding, it is important to consider that each lymphocyte in the simulation bears a receptor (called BCR for B cells, TCR for both THs and CTLs), and APCs contain a definition of HLA class I and II molecules with which they are equipped. Moreover, interactions among cells can be either nonspecific (e.g., macrophages engulfing antigens) or specific. Specific interactions must be accounted for when antibodies meet antigens or when T-cells interact with other cells presenting foreign peptides on their MHC.

An antigen is defined by a part or by the entire proteome, i.e., a set of protein sequences imported via one or more FASTA files (http://www.proteomecommons.org/data/fasta/fasta.jsp).

We make use of the following definitions. Let 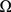 be the set of AA symbols, that is, 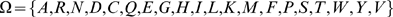. 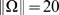 indicates the number of elements in 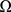. Let 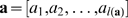 (where 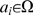) represent a contiguous stretch of AAs, where 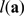 indicates the length of the sequence and 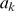 the 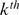 AA in the sequence.

In the following, we use 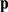 to indicate peptides, whereas we use 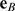, 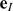, and 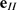 to indicate epitopes for B cells, CTLs, and TH cells, respectively.

### Class I epitopes

**Figure 4:**
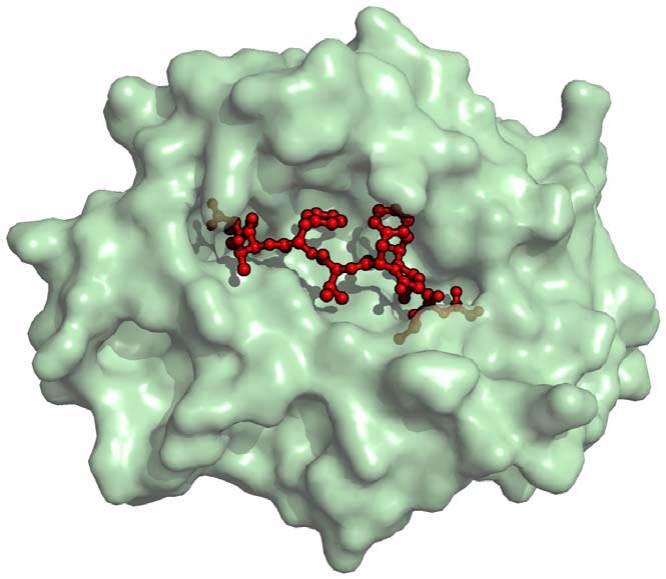
Three-dimensional representation of an MHC class I molecule (in green) complexed with a peptide (in red). The structure has accession number 1OGA in the Protein Data Bank (www.pbd.org).

Class I-type epitopes are linear sequences of 8 to 11 amino acids that are processed from any protein of the pathogen via the process described in section. Each MHC class I molecule, whose total number surpasses the thousands of alleles to date [5], is characterized by a specific binding motif that is possible to “decode”. For the vast majority, the motif length is nine AAs long (Figure 4). Class I T cell epitope prediction methods rely on machine learning techniques. In previous work, we showed that quantitative NNs, which had been trained to predict binding versus non-binding peptides, are superior to the conventional NNs [72]. Furthermore, quantitative NNs allow the straightforward application of a *query by committee* (QBC) principle, in which particularly information-rich peptides can be identified and subsequently tested experimentally. Iterative training based on QBC-selected peptides considerably increases the sensitivity without compromising the efficiency of the predictions [75].

Because we want to handle generic proteins, the portions of a protein that trigger an immune response must be identified. To this purpose, we use the binding motif matrices generated from the NN methods described in [5]. In short, we rank a set of one million randomly selected natural peptides from the human genome using the NN method; the top one percent of the peptides flagged as binders are used to generate a binding motif, i.e., a 9 by 20 matrix. The matrix is calculated using sequence weights, and is corrected for low counts [4], [76]. The average score of the low-scoring binders in the top one percent is set as a threshold value for the matrix. This threshold is then used to discriminate between epitopes and non epitopes as follows.

The propensity is calculated as 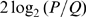, where 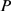 is the probability of finding a given AA at a given position, and 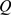 is the probability of finding that AA in any protein in general. These propensities are computed for each of the nine positions on a potential epitope, and give the propensity for each of the 20 AAs. Each matrix represents an approximation of the underlying NN, but the matrix representation is computationally much faster than the computation of the NN directly. Each row of the matrix represents a position in the 9-mer, and the columns correspond to the scores for that specific AA (an example is given in Table 2).

**Table 2:** Scoring matrix for allele A*0301.

Position	1	2	3	4	5	6	7	8	9
A	0.9	−0.9	0.9	0.8	−0.5	0.1	−0.1	0.1	−5.7
R	2.2	−6.8	−0.8	0.7	0.4	−0.2	−0.9	−0.3	−0.1
N	−2.9	−4.7	0.9	0.5	−0.7	0.9	−1.0	0.1	−5.4
D	−6.7	−7.3	−2.0	−0.6	−1.3	−1.6	−2.6	−1.8	−6.0
C	−3.2	−6.2	−2.7	−1.4	−2.7	−2.4	−2.6	−4.6	−8.1
Q	−1.6	0.3	−1.5	0.6	0.5	0.4	−1.4	−0.4	−4.0
E	−5.5	−6.7	−3.1	−0.1	−0.2	−1.2	−2.8	0.3	−4.5
G	−0.1	−5.6	−1.2	−0.5	−1.2	−0.8	−0.3	−0.6	−6.8
H	0.2	−7.1	−0.8	−0.2	−0.2	0.1	−1.4	−0.2	−3.5
I	1.0	2.3	−0.4	−1.2	−0.4	0.5	0.4	−1.0	−7.5
L	0.0	3.0	1.0	−0.8	−0.3	0.4	1.4	0.6	−7.3
K	2.5	−6.1	0.0	0.8	0.5	−0.8	−1.3	0.1	7.4
M	1.1	3.1	1.6	−0.5	0.5	−0.2	1.2	−1.8	−6.4
F	−1.8	−3.4	2.4	0.3	0.3	0.4	1.5	1.3	−6.6
P	−5.6	−7.1	−4.2	0.3	0.5	0.6	−0.1	1.0	−6.2
S	0.9	1.2	0.8	1.2	1.2	1.3	1.6	0.7	−5.4
T	−0.3	2.3	−2.2	0.1	0.3	0.2	0.4	0.2	−5.9
W	−7.1	−7.1	0.6	−0.6	0.9	−0.2	0.9	−0.4	−6.6
Y	−1.7	−5.1	2.8	0.3	1.2	0.1	0.2	2.0	3.1
V	0.3	1.8	−2.2	−1.5	0.1	−0.3	−0.4	−1.3	−7.3

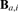
 is the matrix entry corresponding to 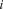 position (columns), 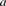 AA (row). Positive numbers indicate that the given AA is favored (often seen) at that position and negative ones that it is not favorable (unlikely).

For a given 9-mer 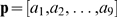, 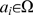, the sum of the values at each position in the scoring matrix gives a score. That is, let 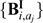 with 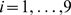 and 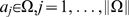, be the matrix of a specific class I MHC allele. The score of a generic 9-mer peptide 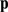 is given by
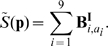
(1)


### MHC class I peptide detection

We next describe how the scoring matrices for alleles are used in the simulation. For an antigenic molecule 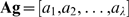, (we assume 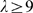), all possible peptides of the protein are found by taking a sliding window of length 9, that is, all possible 9-mers are
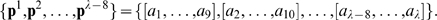
For each 9-mer 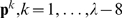, eq(1) computes the score of the peptide 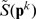. Of all possible 9-mers, those for which 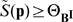, where 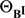 is the allele-specific threshold, are considered epitopes. Hence, the *epitope profile* is
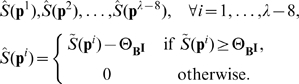
(2)


Note that 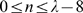, that is, 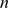 can also be zero, meaning that no epitopes are found in the antigen AA sequence. The threshold 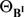 is computed as follows: for the set of peptides used to compute the matrix for each allele, the matrix predictions for binding affinity are calculated. Next, we extract the 1% strongest binders, i.e., those with a high affinity for the MHC. Some of these 1% have a lower binding affinity than others. We consider the 10 weakest binders of this subset to have a low-end binding affinity, and we average these binding scores to get 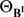. We assume that in a random set of peptides, around 1% have a binding affinity below 500 nM for the MHC and are considered binders [72], [77]. An example is provided in Figure 4.

### Class II epitopes

Class II-type epitopes are presented only on the surface of APCs. For MHC class II epitope detection, we resort to the same methodology used for class I. It is known that class II epitopes have lengths that vary by up to 30 AA [78], [79]. An analysis of *all known class II human binders* from the EPIMHC database reveals that the average class II epitope is 16 residues 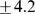 in length (note that the total number of epitopes found to bind human MHCs was 2503 as of March 2008) [78]. Still, the binding core of the peptides presented by the MHC can be reduced to a 9-mer with flanking regions of variable length as demonstrated by Nielsen [80]. This means that MHC class II epitope detection can rely on the same principles as class I epitope detection. To this end, in analogy to section, we created a set of matrices, 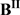, able to score any given 9-mer for each allele covered by the NN method.

### MHC class II peptide prediction

Formally, we compute the score for each possible 9-mer 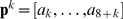 with 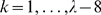 of the antigen AA string 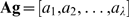 in a manner similar to that described in eq(1). That is, we compute the epitope profile as
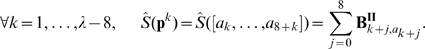
(3)Then, we compile the potential epitopes (meaning that they will be checked for actual binding with the MHC class II), which are the 9-mers scoring above a certain threshold 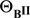,
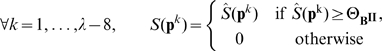
(4)into a list of class II epitopes. We call these AA strings epitopes, indicated by 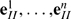. Note that, once again, 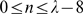. The threshold 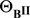 is computed in the same fashion as 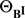.

MHC class II binding prediction is problematic. The 9-mers form only the core of the binding peptide, the variability in alleles is much wider than in class I alleles, and the available prediction methods do not match the prediction capabilities of MHC class I predictors [69]. To remedy these problems, we focused on a limited set of MHC class II alleles for which good predictions exist, and selected those available in the TEPITOPE method [81].

### B cell epitope

The prediction of discontinuous B cell epitopes is still a major challenge in vaccine design, and is difficult for two reasons: first, available data on discontinuous epitopes in different antigens is scarce compared to the available data on linear epitopes; second, few antigens are completely annotated with respect to multiple discontinuous epitopes in a single antigen. The presence of epitopes that are not annotated in the data set increases the difficulties associated with assessing the performance of prediction algorithms.

Due to these difficulties, the majority of prediction tools available for B cell epitopes are based on linear prediction methods. These are limited to continuous stretches of protein sequences that may, in the end, be combined to form one or several conformational epitopes.

Most tools available for the prediction of linear B cell epitopes use propensity scale methods. These methods assign a propensity value to each AA in the queried protein sequence based on knowledge of the AAs physical and chemical properties. Propensity scales have been developed based on antigenicity, hydrophilicity, inverted hydrophobicity, accessibility, and secondary structure. As part of the development of a new prediction method for linear B cell epitopes, we tested all such scales for their ability to predict B cell epitopes in an annotated data set taken from Pellequer *et al.* [82]. It turns out that the propensity scales of Parker (based on hydrophilicity) [83]. and Levitt (based on the secondary structure) show better performance compared to other scales.

For the present work, we decided to use the Parker hydrophilicity method rather than the BepiPred method because the former is simpler and the performance gain using BepiPred is marginal [66].

### B cell epitope detection

The Parker propensity scale [83] is used to find B cell epitopes in a generic antigenic sequence. The Parker propensity scale of AA 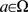 is indicated by 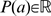 (see Table 3).

**Table 3:** Parker's propensity scale (from [83]).

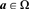	R	D	E	K	S	N	Q	G	P	T
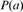	0.87	2.46	1.86	1.26	1.50	1.64	1.37	1.28	0.3	1.15

A	H	C	M	V	I	L	Y	F	W
0.03	0.30	0.11	−1.41	−1.27	−2.45	−2.87	−0.78	−2.78	−3.00

For each AA 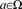, the propensity is indicated by 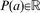.

To calculate the propensity of an AA 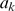, we use the average of the propensities of the AAs in a window ranging from position 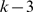 to 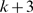. This smoothing window size has been shown to give more accurate B cell epitope predictions [66] because B cell epitopes are generally larger than a single AA. Let 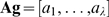 be the antigenic sequence. We compute the score with a smoothing window of seven AA, meaning that we consider three residues on either side of the AA in question. We then create a score profile for the sequence, 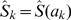, 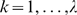, as follows:
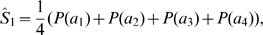


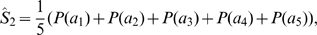


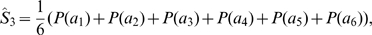


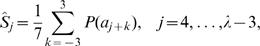


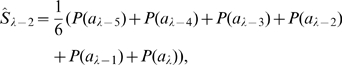


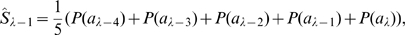


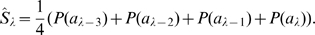
The profile is used to discriminate between residues that are likely to be part of an epitope and those that are not. We use a minimum score threshold 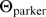


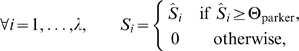
where 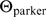 is 0.7. This value gives the best correlation between predicted and real epitopes in the dataset used in [66]. Finally, we label only contiguous regions of AAs, with profiles above the threshold and lengths that are at least four, as possible B cell epitopes. We call 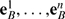, 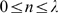 the B cell epitopes found.

### Combined model

The simulation of the full sequence of system events, from antigenic injection to the immune response, proceeds via antigen recognition by lymphocyte receptors.

### The contact potential of Miyazawa and Jernigan

There are no prediction tools available for describing specific binding among BCRs, antigen epitopes, TCRs, and generic MHC-peptides (both class I and class II). Therefore, we had to define, in C-ImmSim, a generic contact potential among AA sequences to be used in those cases.

The work performed by Miyazawa and Jernigan on protein energy potentials [84] provides us with a method for assessing the chances of direct interactions among proteins in the simulation. The protein–protein potential concept was derived from the analysis of 3D structures in which the relative position of AAs were determined. The contact potential matrix estimated by Miyazawa and Jernigan reflects the entropy between two residues. A low entropy means that the pair of residues has low energy and, therefore, that interaction is possible.

The contact potential defined between two AA strings is, thus, based on the Miyazawa-Jernigan score. In the simulation, this measure is used both when a BCR meets an antigen and when a TCR meets an MHC-peptide complex. For the case of BCR, we use a mean field approach, meaning that we assess the potential of the whole BCR against the B cell epitope.

Let 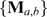, with 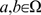, be the matrix found in [84]. If 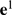 is a BCR and 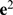 is a B cell epitope, then we use the following formula:
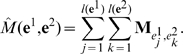
(5)


For T-cell recognition, the procedure is different because it requires the definition of a class of specific contact matrices 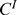 and 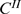 for class I and class II, respectively.

**Figure 5:**
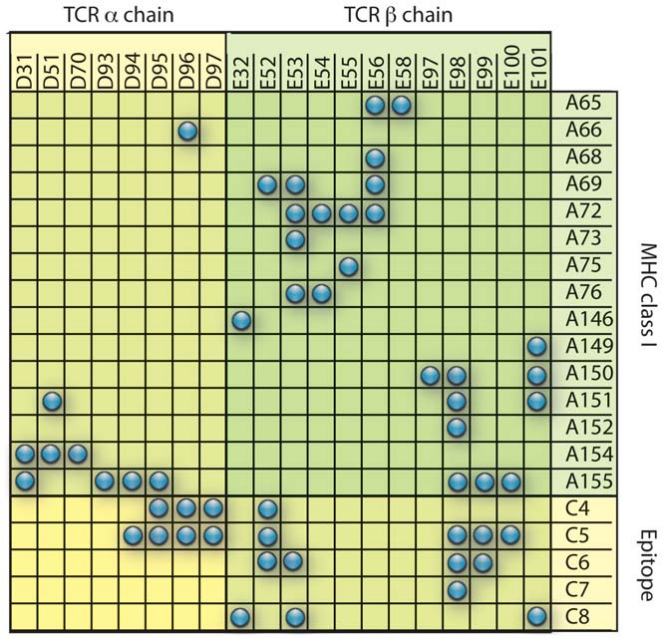
The contact matrix used for class I presentation and TCR binding. Labels on the axis represent positions on the peptide-MHC complex that are in contact with the TCR chains 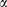 and 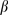. The matrix was derived using the structure indexed under the reference 1OGA in the PDB database. Labels on the columns report the position indices for the residues in the two TCR chains as they are numbered in the PDB file (chains E and D respectively). Rows: Labels report the position indices for the MHC residues and the peptide (chains A and C, respectively, in the structure file). A blue dot means that the pair of residues in the row/column are within 5 Å distance, and are considered to be in contact. Otherwise, they are not. In the program, this matrix is coded with ones (blue dot) and zeros (no dot).

We precomputed the contact matrices from known protein 3D structures found in the Protein Data Bank (www.pdb.org) taking residues that i) are within a distance of 5 Å and, ii) show contacts between the MHC-epitope complex and the two chains (heavy and light) of a bound TCR. The distance of 5 Å was selected because most crystal structures with experimentally verified B cell epitopes show that the residues on the antibody in contact with an epitope lie within a 5 Å radius. We extend the use of this value to the minimum distance needed between residues for molecular interaction. By using the solved structures, it is possible to determine which residues on a TCR bind to the MHC and peptide, and which should be considered to be in the MHC–peptide complex. The contact matrix derived for class I binding is represented in Figure 5.

Therefore, if 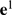 is a TCR, 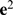 is a MHC-peptide complex, and 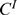, 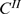 are the contact matrices used for class I and class II respectively, the binding affinity between the residues is
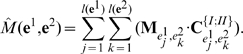
(6)


Now, in order to determine effective thresholds for the interaction strengths defined above, in eq(5) and eq(6), we observed that, given two randomly chosen AA strings 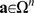 and 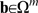, (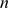 and 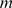 also taken at random), the score 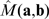 follows a Gaussian distribution with average 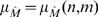 and standard deviation 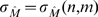. Therefore, we pre-estimated those values of 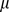 and 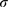 for a wide range of 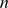 and 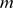, and we defined the normalized score as follows:
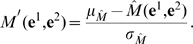
(Note that 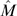 is negative.)

Next, we select those with a normalized score above threshold 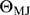 as potential binders (i.e., positive probability), i.e.,
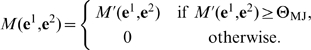
(7)


The threshold value of 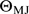 determines the number of reactive clones and was estimated to 0.075 so that in a typical immunization experiment, antigen clearance is obtained in a time frame of a few days. We use 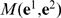 of eq(7) as the probability to decide if 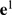 binds 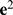.

### Putting all parts together: The simulation of immune recognition

The simulation follows the same procedure as in the original bit-string version [40], with the significant difference being that antigen recognition and binding rely on the epitope prediction methods described above. In the new model, we represent pathogens at the protein level by their AA sequences, which means that we implicitly account for only transcribed DNA. The host's genotype is defined by a set of four MHC class I and class II alleles.

The space volume is populated with an initial number of entities. Lymphocytes are generated with a random AA receptor of length 48 for BCRs and 32 for TCRs.

The sequence of events culminating in the immune response (either humoral, cytotoxic, or both) is described in the following.

1. The 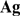 represented by one or more AA strings is injected;
  - the B cell epitopes 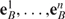 are probed. Here we use the method described in section;
  - for each MHC of class I and II, the T-cell epitopes 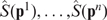 are found and scored (see section and section);
2. Phagocytosis by antigen processing cells;
  - M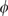 s and DCs perform unspecific phagocytosis of 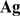;
  - B cells must recognize, with their B cell receptor BCR, at least one epitope of the 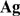. Phagocytosis happens with a probability 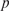 defined as follows:
    Given the precomputed B cell epitopes 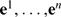, we calculate, for a B cell receptor BCR, the score 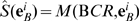 by means of the MJ method, and normalize those scores as described in eq(7) to get 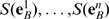.
      Finally, the probability that a B cell will recognize at least one of the epitopes is calculated as 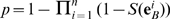, that is, the probability for the BCR to match at least one epitope of the antigen;
3. Antigen digestion by APCs. Once an APC (M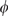, DC, and B cell) has internalized the antigen, it is processed as follows:
  - Because the epitopes 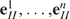 have been determined as described in section, we can randomly select. This selection is performed by means of the *random wheel selection* procedure: draw a number 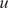 between 0 and 1 with a uniform probability distribution and select 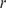 if 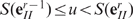. One epitope 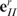, with a probability that is given by the normalized score 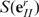;
  - Analogously to the endocytotic digestion, endogenous digestion takes place in cells that are infected by a virus. In this case, the epitopes 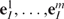 are found by using the method described in section;
**Building the MHCI-peptide sequence:** each infected cell bears a set of two A and two B alleles.
    This implies that each protein from the pathogen is processed at most four times during the discovery process for class I epitopes. After processing the antigen protein, each cell presents, on its surface, one randomly chosen epitope with a probability that is proportional to the score of that epitope divided by the sum of scores of all found epitopes. This choice reflects the competition for MHC molecules among the protein fragments produced by the proteasome. Inside a single infected cell, antigen peptides are processed so that they bind one MHC class I molecule. Because we allow cells to display only one MHC peptide molecule per time step on their surface, we have to choose the display protein from within the haplotype (i.e., the four available, two A- and two B- alleles). This is performed by random selection at each time step. The procedure computes the epitope profile 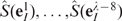 as described in eq(2), then normalizes it as follows:
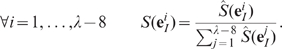
(8)The normalized profile is used to select, from the probability distribution 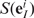, the epitope 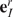 to be presented on the surface of the cell.
    This complex is then used to compute the matching score against the cytotoxic T cell receptor (see section).
4. The APC shows the MHCIIpep (the complex formed by an MHC class II and a nine AA-long peptide) on its surface for TH-TCR recognition. This recognition makes use of the score defined in eq(7);
5. Humoral response
  - Stimulated B cells start cloning and differentiating into long-lived memory cells and antibody-producing plasma cells;
  - Plasma cells secrete antibodies;
  - Antibodies bind antigens' epitopes;
    to compute the affinity between antibodies and the antigen, we follow the same procedure as the one applied for antigen recognition by B cells, (section and section);
    an immunocomplex is formed by the combination of an antibody and an antigen;
6. Cytotoxic response;
  - For infected cells showing MHCIpep (a complex formed by an MHC class I and a 9-mer) on their surface, recognition of CTLs via their TRC is performed using the Miyazawa-Jernigan potential between the MHCIpep and the TCR. The normalized score (eq(7) in section) is used as the probability of binding;
  - Upon successful recognition (i.e., binding), cytotoxic cells kill virus-bearing cells and start cloning.

### Thymus education of T lymphocytes

As mentioned, we filter randomly created T cell receptors by means of a procedure that mimics the positive and negative selection of immature thymocytes in the thymus gland. This reflects the *clonal deletion theory* proposed by Burnet, according to which self-reactive lymphoid cells are destroyed during the development of the immune system to prevent autoimmunity.

**Figure 6:**
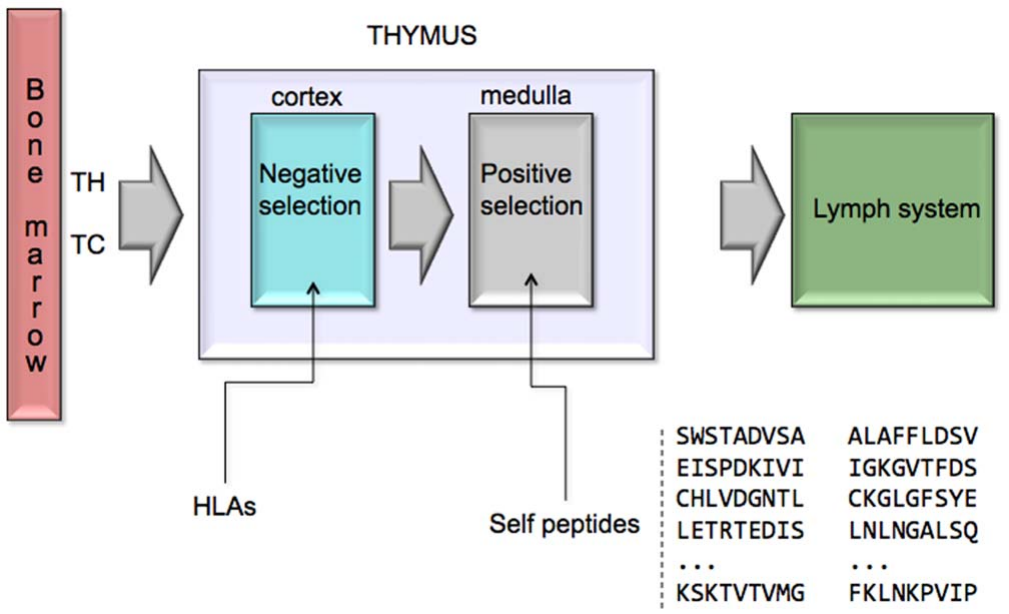
The two-layer filter realized by the thymus to eliminate auto-reactive T lymphocytes. T-cells develop *self tolerance* during negative selection, whereas they are eliminated as useless during positive selection.

In C-ImmSim, the thymus is modeled as a *two-layer* filter (see Figure 6), and the same procedure for detecting antigen peptides is used to differentiate self peptides from proteins that represent *the self*. This process allows T-cells to develop *self tolerance* (in the negative selection) while eliminating useless cells (positive selection). The self is defined by specifying a random set of naturally occurring 9-mers extracted from the human proteome. These peptides are the same as those that have been used to compute the matrices for the different MHC molecules.

In practice, we allow a T cell to enter circulation (i.e., to reach a secondary organ as a mature thymocyte) with a probability given by the product of the probability of being positively selected and the probability of being negatively selected,
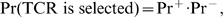
(9)with
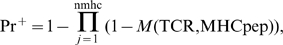
(10)where 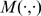 is the Miyazawa-Jernigan contact potential calculated as in eq(7), the only difference being that residues in contact with the MHC and the TCR are taken into account, because there is no peptide attached to the MHC at this stage.

Negative selection is performed according to the following procedure: for each 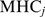 (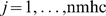) and for each self AA string 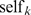 (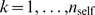),

- compute the sequence profile of the 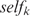 with respect to the 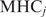, as described in section and section, according to whether the T is a helper or a cytotoxic T cell;
- randomly choose a peptide and create an 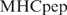 string;
- compute the Miyazawa-Jernigan contact potential 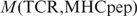.

Finally, the probability that a T cell survives negative selection is
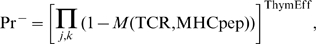
(11)where the MHC molecule is composed of 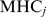 and the chosen peptide of the 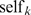. The exponent ThymEff is required because we treat the thymus as if it were composed of ThymEff sub-layers (by simulating multiple encounters with each thymic cell receptor).

### Parameters of the model

The simulator accepts, as input, the definition of the antigen AA sequence (in the form of a FASTA file), the matrices defining the binding motifs for the haplotype (four matrices for class I, two HLA-A and two HLA-B, as well as two matrices for class II, as explained in section and section), and other variables that are in part derived from the literature and in part are free parameters used to tune the system. Most of the parameters of this version of C-ImmSim are the same with respect to the previous bit-string version. The parameters are described in http://www.iac.cnr.it/filippo/parameter-page.html. The main difference consists in the fact that, now, all clonotypic receptors, peptides, and epitopes are represented by strings of AAs. Moreover, the definition of the HLAs is now given in terms of affinity matrices rather than in bit-strings..

In the following experiments the self is given as a random set of naturally occurring 9-mers extracted from the human proteome. Since we are not focusing on studying th emergence of autoimmunity diseases, we arbitrarily take 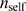 = 50 and ThymEff = 5.

As output, the simulator produces a set of files corresponding to population data (both total number of lymphocytes and the division between clonotypes, cytokines, and antibody concentrations per lattice point) plus Logo files [85] of lymphocytes at certain time steps.

**Figure 7:**
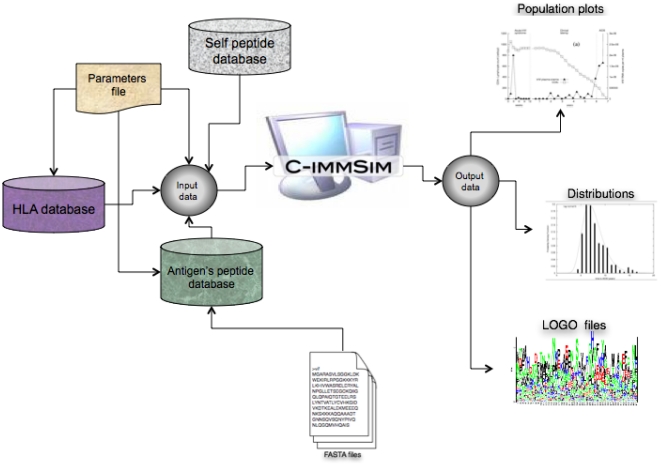
The overall architecture of the simulation tool. The definition of HLAs is given by means of the precomputed matrices, as described in sections and. Moreover, we select the pathogen as a collection of peptides from a database of FASTA files. The output of the simulator consists of a set of ASCII or binary files describing the state of the system at each time step. From the files, various statistics can be extracted.

The overall architecture is depicted in Figure 7.
